# Modulating somatosensory alpha oscillations using short-period transcranial alternating current stimulation

**DOI:** 10.1162/imag_a_00531

**Published:** 2025-04-07

**Authors:** Vaishali Balaji, Alfons Schnitzler, Joachim Lange

**Affiliations:** Institute of Clinical Neuroscience and Medical Psychology, Medical Faculty and University Hospital Düsseldorf, Heinrich Heine University Düsseldorf, Düsseldorf, Germany

**Keywords:** transcranial alternating current stimulation, magnetoencephalography, alpha oscillations, somatosensory cortex

## Abstract

Transcranial alternating current stimulation (tACS) appears to modulate neuronal oscillations at the frequency of stimulation. Longer periods of stimulation with tACS (10–40 min) have shown to produce persistent changes, especially in alpha power (~8–12 Hz), whereas the efficacy of shorter periods of tACS (1–8 s) is less known. Thus, we investigated whether short periods of tACS applied to the somatosensory cortex elicit changes in alpha power following stimulation. With this aim, during simultaneous acquisition of MEG, we administered tACS and control (no-tACS) on separate days. We applied short trains of stimulation for durations of 10 s and 30 s at an individually adapted stimulation frequency (ISF). Each stimulation train was followed by a 15 s interval. We calculated power changes in the post-stimulation intervals, relative to a baseline period, and the resulting Δpower was used to statistically test the difference between tACS and control conditions. We found significant elevations in power at ISF following tACS compared with control. The extent of this effect spanned bilaterally over the somatosensory and frontal regions. While the observed increase in power was most prominent around ISF (i.e., in the alpha band), power modulations were also observed in the beta-band. When comparing the two stimulation durations, 10 s of tACS produced greater increases in power (at ISF) than 30 s of tACS. This study validates that 10 s of tACS produces robust elevations of power in the somatosensory cortex at ISF, thereby establishing its potential for use in future studies.

## 1 Introduction

The use of electrical stimulation to study the cerebral cortex has a long-standing history in neuroscience (Fritsch & Hitzig, 1870). A non-invasive electrical stimulation method that has gained traction in recent years is transcranial alternating current stimulation (tACS). This method involves the application of sinusoidal currents to the scalp with the aim of modulating neuronal oscillations, and in turn, functionally relevant behaviour. Animal studies propose that tACS acts via entrainment: a mechanism by which oscillations synchronise to the phase and frequency of the externally applied current (Fröhlich & McCormick, 2010;Ozen et al., 2010). Whereas, effects of tACS that persist beyond the period of stimulation have been attributed to spike timing-dependent plasticity (Schwab et al., 2021;Vossen et al., 2015;Wischnewski et al., 2019;Zaehle et al., 2010). An opposing view is that oscillations are indirectly entrained via stimulation of peripheral nerves on the scalp (Asamoah et al., 2019). The exact mechanisms underlying the interaction between tACS-induced electric fields and the targeted neuronal oscillations remain a matter of contention (Vogeti et al., 2022).

tACS is often targeted at oscillations in the alpha range (~8–12 Hz) given its relevance in the parieto-occipital region (Buzsáki et al., 2013;Hari et al., 1997) for perception and cognition (Baumgarten et al., 2016;Jensen & Mazaheri, 2010;Klimesch et al., 2007;Samaha & Postle, 2015;Van Dijk et al., 2008). Modulation of alpha oscillations affects perception (Battaglini et al., 2020;Cecere et al., 2015;Fiene et al., 2022;Harada et al., 2020;Kemmerer et al., 2020), motor learning and consolidation (Schubert et al., 2020), and functional connectivity between cortical regions (Gundlach et al., 2020;Helfrich, Knepper, et al., 2014;Schwab et al., 2019). Typically, these studies used long stimulation durations (of 10–40 min), yielding long-lasting power enhancements that persist for several minutes post-stimulation (Helfrich, Schneider, et al., 2014;Kasten et al., 2016;Neuling et al., 2013;Zaehle et al., 2010).Stecher and Herrmann (2018)found no evidence of power modulation following blocks of 1 to 10 min of stimulation, which was attributed to low illumination and a mismatch between stimulation frequency and endogenous alpha frequency. Besides this study, there have been no systematic investigations of the duration-dependent effects of tACS. Therefore, it is unclear whether longer periods of stimulation are beneficial for eliciting electrophysiological after effects.

Short-intermittent tACS offers the potential to manipulate power immediately before stimulus presentation, that is, within task-relevant time scales. However, the reported effects of short stimulation periods (in the range of seconds) are not well established.Vossen et al. (2015)found that stimulation at individual alpha frequency for trains of 8 s, but not 3 s, increased alpha power compared with sham (replicated byStecher et al., 2021). One study demonstrated an increase in stimulus-evoked power using 1 to 1.8 s stimulation trains, with a fixed frequency of 7 Hz (Stonkus et al., 2016).Ruhnau et al. (2016)showed that 2 s of stimulation amplified steady-state responses, but only when frequency of the flickering stimulus and tACS matched. Overall, shorter train durations may not enhance power (Strüber et al., 2015;Zarubin et al., 2020). In addition to stimulation duration, frequency and intensity of applied current differ between the above-mentioned studies; therefore, it is difficult to pinpoint the reason for the reported differences in results. Furthermore, most studies targeted visual alpha with complementary electrode placements (typically Cz and Oz). While augmenting alpha oscillations should affect perception in visual and somatosensory modalities alike, only a few studies have shown concomitant alterations in tactile perception (Gundlach et al., 2016;Saito et al., 2021;Sliva et al., 2018), whereas others reported null findings (Manzo et al., 2020;Wittenberg et al., 2019). Notably, only one study tested for increments in power after stimulation (Sliva et al., 2018). Given the paucity of evidence for power modulation in the somatosensory domain, our goal was to assess the suitability of short-period tACS for prospective studies.

In this study, we acquired magnetoencephalography (MEG) simultaneously during tACS. We applied tACS over the right primary somatosensory cortex (S1) at the individuals’ alpha frequency. In contrast to most tACS studies, we employed a ring electrode montage for more focal stimulation (Datta et al., 2008). Additionally, we anaesthetised the region on the scalp underneath the electrodes to control for indirect entrainment via transcutaneous stimulation. We administered tACS for periods of 10 and 30 s, followed by a post-stimulation interval. Since trains of 1 to 6 s are unsuitable, we chose 10 s as a starting point. We included an additional train duration (30 s), as longer stimulation durations revealed promising results. To assess after effects of tACS, we compared power in the post-stimulation intervals to resting-state baseline and a control condition.

## 2 Methods

### 2.1 Participants

Twenty-six volunteers participated in the study. A sample of 20 to 24 participants was deemed fit based on studies byVossen et al. (2015)andStecher et al. (2021). Two participants were excluded from the analysis: one due to poor performance on the vigilance task and the other because of a broken head position indicator (HPI) coil. In total, 24 participants were included in the final analyses (11 female, age 25.33 ± 2.81 [mean ± SD] years). We excluded one block (in participant No. 15), as we failed to record the baseline period. Participants had normal or corrected-to-normal vision and met standard inclusion criteria for MEG experiments. No participant had a history of epilepsy or other neurological or psychiatric conditions.

The study was approved by the ethics committee (ethics vote no. 4965) of Heinrich Heine University Düsseldorf, Germany. Participants gave written informed consent and received a monetary compensation of 10€/h.

### 2.2 General procedure

Participants attended two experimental sessions conducted on separate days. We administered tACS in one session and the other session served as a control. Prior to this, we acquired anatomical T1-weighted MRIs using a 3T Siemens scanner (Siemens Magnetom Tim Trio 3T, Siemens, Germany).

**Fig. 1.**
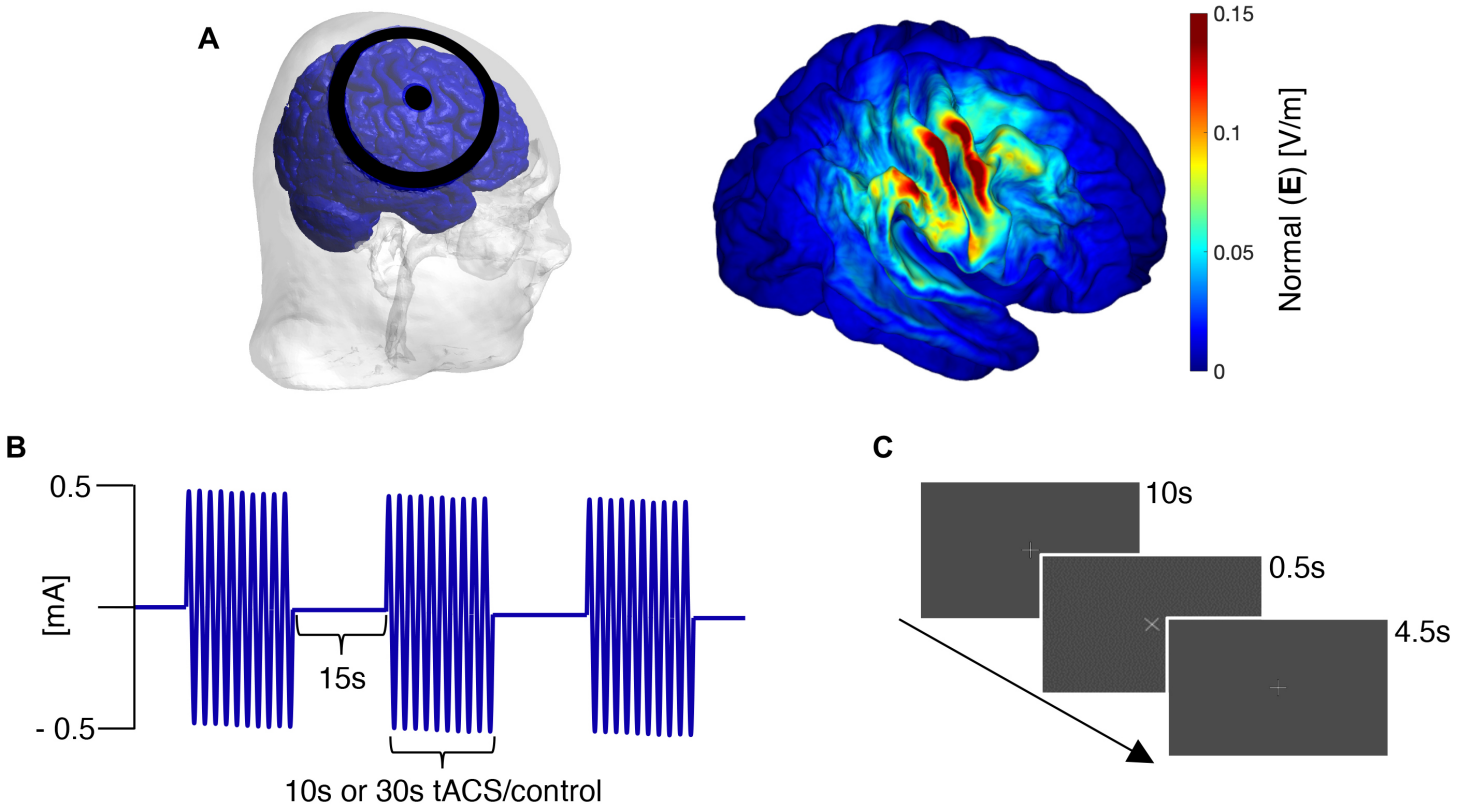
Electrode montage and stimulation protocol. (A) Illustration of electrode placement and simulation results. A ring electrode montage was attached to the scalp corresponding to right S1 (left panel). Grand mean of the normal field component (right panel) projected in FsAverage space. The maximum electric field strength was approximately 0.14 V/m. (B) Stimulation protocol. All participants underwent 10 s*tACS,*30 s*tACS,*and control sessions in separate blocks. Train duration was 10 s in one*tACS*block and 30 s in the other. Trains were followed by a 15 s interval of no stimulation. tACS was applied at 0.5 mA (zero-to-peak) amplitude. No stimulation was applied in the control condition. (C) Schematic representation of a single 15 s post-stimulation interval. A fixation cross was present through the entire duration of the experiment. In any 9 of 20 inter-stimulation intervals, the fixation cross rotated for 500 ms. Participants were instructed to press a button to indicate seeing the rotation. After 4.5 s, the next stimulation train started.

The experimental procedure was identical in tACS and control sessions. First, we individually localised the right primary somatosensory cortex (S1) using the Visor2 neuro-navigation system (ANT Neuro, Netherlands) and participants’ MRI. Next, we applied a topical anaesthetic (Anesderm cream; Prilocaine 25 mg/g and Lidocaine 25 mg/g) corresponding to the site of S1 on the scalp (Fig. 1A, left). About 1 h after applying the cream, we attached the tACS electrodes to the anaesthetised region using Ten20 conductive paste (Weaver & Co., USA). At the end of each session, participants reported feelings of “alertness,” “contentedness,” and “calmness” on Bond and Lader’s Visual Analogue Scale (BL-VAS;Bond & Lader, 1974). Participants also indicated if they felt any sensations on the scalp during the experiment (with yes/no) and rated their level of confidence (on a scale of 1 to 10) for having received tACS stimulation.

### 2.3 Stimulation protocol

Participants were seated upright in a dimly lit magnetically shielded room while we acquired MEG simultaneously during tACS or no-tACS (control session). We administered tACS via a ring electrode montage (Fig. 1A, left), with a small circular electrode (Ø: 2 cm) and a larger concentric electrode (outer Ø: 11 cm; inner Ø: 9 cm; NeuroConn, Germany). The stimulation currents were generated by a battery-operated stimulator (DC Stimulator, NeuroConn, Germany), whereas the timing of stimulation was controlled by presentation (Neurobehavioral Systems, USA). We maintained electrode impedance below 15 kΩ. Simulations of electric fields were set up using SimNIBS 4 MATLAB functions (Thielscher et al., 2015). We used Gmsh to visualise the position of the electrode (Geuzaine & Remacle, 2009). We averaged the normal (or strength) of the electric field across participants (Saturnino et al., 2018), and projected the results in FreeSurfer’s FsAverage brain template (Fig. 1A, right).

We administered two stimulation blocks per session. In each block, following a 2-min resting-state baseline measurement, we applied 20 trains of stimulation in an intermittent fashion (Fig. 1B; adapted fromVossen et al., 2015). The duration of each stimulation train was 10 s in one block and 30 s in the other, and we counterbalanced the order of blocks between participants. Each stimulation train was followed by a 15 s inter-stimulation interval. In the tACS session, amplitude of the current was kept constant at 1 mA (peak-to-peak), whereas the frequency of stimulation was individually adapted (see below section). No stimulation was applied in the control session.

To maintain attention, participants performed a vigilance task (Fig. 1C). We projected a white fixation cross on a black background (PT-DW700E, Panasonic, Japan) at a distance of ~57 cm from the participant. The fixation cross (of 2°visual angle) rotated 10 s after stimulation offset by 45° for a short period of 500 ms. The rotation was triggered during any 9 out of 20 inter-stimulation intervals per block. We instructed participants to press a button using their right index finger within 2 s of the rotation.

### 2.4 Individual stimulation frequency (ISF)

To determine the frequency of stimulation, we estimated alpha peak frequency for each participant from a 2-min resting-state measurement. We tapered the data with a single Hanning window and applied a Fast Fourier Transformation for frequencies from 4 to 40 Hz in steps of 1 Hz. We averaged spectral power across a fixed set of right parietal channels (“MEG2412 + 2413,” “MEG2422 + 2423,” “MEG2432 + 2433,” “MEG2442 + 2443,” “MEG2612 + 2613,” “MEG2622 + 2623,” “MEG2632 + 2633,” “MEG2642 + 2643”). From this averaged spectrum, we determined the peak frequency, that is, the frequency with maximal power, within a broad alpha range (7–14 Hz;Table S1).

### 2.5 MEG data acquisition and preprocessing

We recorded neural activity using a 306-channel MEG system (MEGIN Oy, Finland) at a sampling frequency of 1000 Hz. We used the Polhemus Fastrak system (Polhemus, USA) to digitise HPI coils, fiducial markers, and ~50–100 additional points. We recorded electrooculogram (EOG) to monitor blinks and eye movements.

We performed preprocessing and analysis of MEG data offline using the Fieldtrip toolbox (v20210825) (Oostenveld et al., 2011) and custom-made MATLAB scripts (R2019b; The MathWorks, Inc., USA). We first segmented the continuous MEG recording into trials. We segmented the 20 inter-stimulation intervals, succeeding tACS or control, into 6.2 s-epochs; 3.8 s to 10 s relative to stimulation offset (henceforth referred to as post-stimulation trials).

The stimulation device systematically produced two artefacts around 10 ms and 3.8 s after every train of stimulation. We observed a power decrease (relative to baseline), resembling a DC offset, in the period between the two artefacts. Therefore, the first 3.8 s of data were unusable. It is likely that the artefacts are a result of the impedance measurement performed by the stimulator in preparation for the next train of stimulation. We also rejected the last 5 s of every inter-stimulation period to avoid movement-related artefacts due to the vigilance task.

We restricted further preprocessing to 204 gradiometer channels. We applied a band-pass filter of 4–150 Hz and removed the linear trends and mean of every trial. Segments of trials containing artefacts (e.g., SQUID jumps, head and muscle movements) were removed via a semi-automatic artefact detection approach implemented in Fieldtrip. Additionally, we removed noisy channels and trials containing artefacts after visual inspection. We used Independent Component Analysis (ICA) to remove remnant electro-cardio and electro-ocular artefacts.

Finally, we cut the post-stimulation trials into 5.5 s epochs, from 4.2 to 9.7 s, to remove edge effects introduced during preprocessing. Further, we removed the last 10 s of the baseline period to avoid device-related artefacts and obtain a uniform number of baseline trials. The remaining 110 s were then segmented into 20 trials, each 5.5 s long.

### 2.6 MEG source projection

To project sensor-level data into source space, we employed the Linearly Constrained Minimum Variance (LCMV;Van Veen et al., 1997) beamformer technique.

To construct individual grids, participants’ anatomical MRIs were co-registered with their MEG data through fiducial markers and the brain was segmented. From the segmented MRI, we generated a single-shell volume conduction model (Nolte, 2003). Then we applied a regular 3D grid (5 mm resolution) to the MNI template brain (Montreal Neurological Institute, Canada). We computed individual grids by nonlinearly warping participants’ MRI on the MNI template brain, and then applied the inverse of this warp to the MNI template grid. We restricted the individual grid to cortical grid points defined by the AAL atlas (Tzourio-Mazoyer et al., 2002), resulting in 8793 grid points.

We computed the covariance matrix from concatenated baseline and post-stimulation trials. Then, the covariance matrix, the individual grid, and the volume conduction model were used to build LCMV filters. To obtain time series for each grid point, we multiplied these filters with sensor time series data.

### 2.7 MEG time–frequency analysis

We performed time–frequency analysis for all cortical grid points for frequencies from 5 to 40 Hz (1 Hz resolution) by applying a Hanning taper to sliding time windows (with seven cycles per time window; 100 ms step size), followed by a Fast Fourier Transformation.

Subsequently, we adjusted the time–frequency representations (TFRs) of both sessions so that stimulation frequencies were aligned across participants. For each participant, the frequencies ranged from “ISF−2” to “ISF+26” Hz.

### 2.8 Statistical analyses of tACS

For each grid point of a participant, we calculated change in power relative to baseline, (i.e., Δpower) using independent t-tests. Prior to this calculation, we averaged power across all 20 trials for baseline and post-stimulation periods, respectively, and we averaged power across time points for the baseline period to obtain a single power value for each frequency. The resulting Δpower provided a measure of power change following tACS or control:

$$\Delta power_{tACS/control}=\frac{power_{post}\;-\;power_{baseline}}{pooled\;standard\;error}.$$

To investigate Δpower on group level, we performed a non-parametric cluster-based permutation test (Maris & Oostenveld, 2007). In single participants, we first averaged Δpower_tACS_and Δpower_control_over time. Then, Δpower_tACS_for the 10 s block and Δpower_tACS_for the 30 s block were pooled together and likewise for Δpower_control_. On group level, we performed paired t-tests for each of the 8793 grid points between pooled Δpower of tACS and control conditions. If the statistical comparison met an*a priori*defined threshold (p < 0.05), t-values of spatially adjacent grid points were combined to a cluster (minimum of two neighbouring channels). We permuted the data by randomising the condition assignment (tACS or control), and on each permutation, the cluster with the maximum sum of t-values was retained. This procedure was repeated 1000 times, yielding 1000 summed cluster t-values, from which the probability of the observed cluster statistic was determined.

*Post hoc*, we investigated whether the observed increase in power was specific to ISF in the cluster. To this end, Δpower_tACS_and Δpower_control_for the respective blocks were pooled together. We averaged pooled Δpower across grid points in the significant cluster (see previous analysis). We performed a cluster-based permutation test as described above; here clusters were based on temporal and/or spectral adjacency. In a control analysis, we replicated this analysis, but we adjusted TFRs based on harmonic multiples of the ISF prior to calculating Δpower (seeFig. S1).

Further, we qualitatively separated the significant cluster into its frontal and somatosensory counterparts to test for region-specific effects. This separation was based on the spatial location of the grid points, as specified by the AAL atlas. The 10 s and 30 s blocks were first pooled together. Then we averaged pooled Δpower across grid points in the frontal and somatosensory clusters, respectively. We used non-parametric permutation testing to statistically compare tACS and control conditions in the two distinct regions (see above).

Lastly, we tested the effects of train duration (10 s and 30 s) on power at ISF. In single participants, we first averaged Δpower across grid points in the entire significant spatial cluster. On group level, we performed paired t-tests to contrast Δpower_tACS_with Δpower_control_. We then statistically compared the resulting t-values between the 10 s block and 30 s block using the Wilcoxon Rank Sum test.

#### 2.8.1 Binning analysis of stimulation trains

We aimed to evaluate whether short tACS trains were sufficient for enhancing power. We averaged power over trials and across time for the baseline period (denoted below as “avg power_baseline_”). We then calculated power-difference for each trial (n denotes the trial number):

$$Power\;-\;difference_{\;trial(n)}\;=\frac{power_{trial\left(n\right)}-avg\;power_{baseline}}{avg\;power_{baseline}}.$$

Subsequently, we averaged power-difference (at ISF) across grid points in the significant cluster and over time in each participant, resulting in a single power-difference value per trial. All trials, in a given stimulation block, were divided into five equal-sized bins based on trial order (i.e., power-difference values of four consecutive post-stimulation trials were grouped into one bin). We used Friedman’s tests to assess the effect of “Bins” on power enhancement.

#### 2.8.2 Exploratory analysis of rhythmic power modulations

Many participants exhibited rhythmic fluctuations in alpha power across time. Thus, we investigated whether this putative underlying rhythm, termed modulatory power, changed with the application of tACS. tACS may influence fluctuations of power in two ways. On one hand, the magnitude of the fluctuation, that is, how strongly power fluctuates at a given frequency, may be amplified by tACS. In this case, tACS merely amplifies the amplitude of alpha activity, without any changes in the modulation frequency. On the other hand, the regularity of these fluctuations, that is, the occurrence of burst-like activity, may be facilitated by tACS. For this analysis, TFRs of power were computed for a sliding window of 400 ms (in steps of 100 ms). All other steps were as described inSection 2.7. We computed power-difference for each trial by subtracting power of the preceding trial (averaged over time) from power of the current trial (n denotes trial number):

$$Power\;-\;difference_{\;trial\left(n\right)}=power_{trial\left(n\right)}-avg\;power_{trial\left(n-1\right)}$$

The first trial was excluded from this analysis since there is no preceding trial. In single participants, we averaged power-difference over grid points in the significant cluster. We calculated the modulatory power in each trial for frequencies from “ISF−2” to “ISF+26” Hz (in steps on 1 Hz). We first filtered the power-difference values (filters between 0.1 and 2 Hz in steps of [0.1, 0.1, 0.1, 0.1, 0.25, 0.25, 0.25, 0.25, 0.25, 0.25] Hz). The filter frequencies were non-uniform to reduce computational load while maintaining good spectral resolution at lower frequencies. We then applied a Hilbert transformation on the filtered data, extracted the modulatory power by computing the absolute value of the Hilbert transformation, and averaged the values across time. On group level, modulatory power in each tACS block was contrasted with the corresponding control block via cluster-based permutation tests (see foregoing section).

## 3 Results

### 3.1 tACS and control session questionnaires

Five participants reported sensations on the scalp during the tACS session (confidence rating 3.80 ± 2.49 [mean ± SD]), indicating that they were unsure whether tACS was administered. Three participants reported sensations during the control session (confidence rating 5.67 ± 1.53). Across all participants, the mean confidence rating in the tACS session was 4.96 ± 3.34, and 4.56 ± 3.18 in the control session. Confidence ratings across sessions were not significantly different (Wilcoxon Rank Sum test; p > 0.05). Overall, the topical anaesthetic was effective in eliminating peripheral stimulation and blinding the stimulation condition.

On the BL-VAS, participants scored 40.58 ± 16.95 for alertness, 14.25 ± 6.27 for contentedness, and 3.92 ± 2.16 for calmness in the tACS session. In the control session, they scored 37.83 ± 17.76 for alertness, 14.63 ± 6.84 for contentedness, and 4.29 ± 2.41 for calmness. Overall, BL-VAS scores did not significantly differ between the tACS and control sessions (Wilcoxon Rank Sum test; p > 0.05). Further, all participants fulfilled at least a 70% detection rate on the vigilance task in both sessions.

### 3.2 tACS effects on alpha power

**Fig. 2.**
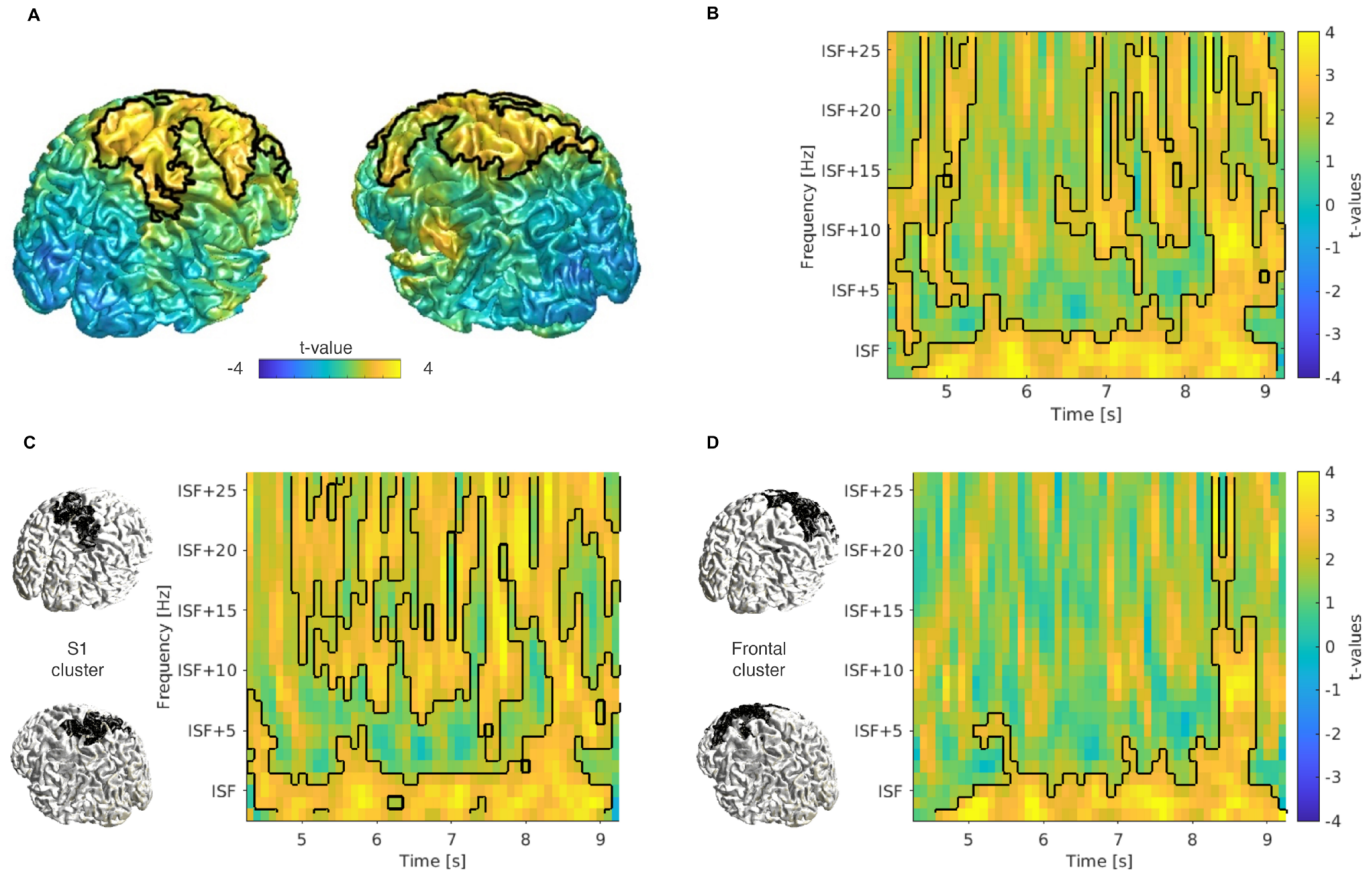
Analysis of power contrast between tACS and control conditions. (A) t-values of Δpower contrast (seeSection 2.8) projected on an MNI template brain. t-values were averaged across the post-stimulation period at individuals’ stimulation frequency (ISF). A higher t-value signifies greater Δpower in tACS than control. We found one significant positive cluster (p < 0.05; outlined in black). (B) Time–frequency representation (TFR) of t-values averaged across channels in the significant cluster shown in (A). TFRs were aligned to ISF before averaging. We found four significant positive clusters (outlined in black). (C) TFR of t-values averaged across channels in the somatosensory part of the cluster shown in (A), as highlighted in the left panel. We found three significant positive clusters (black outlines in right panel). (D) TFR of t-values averaged across channels in the frontal part of the cluster shown in (A), as highlighted in the left panel. We found one significant positive cluster (black outlines in right panel).

An increase in power was observed at individual stimulation frequency (ISF) after tACS compared with control (p = 0.007;Fig. 2A). The power change in the tACS session was 31.68 ± 26.72% [mean ± SD], and 16.43 ± 22.46% in the control session. The cluster extended bilaterally in the somatosensory and frontal regions.

Next, we investigated whether the power increase was specific to the alpha-band. We found four significant time–frequency clusters (p < 0.030;Fig. 2B). The clusters extended broadly across all frequencies, but the power increase was most prominent between ISF and “ISF–2” Hz. In the control analysis, we aligned ISF to its first harmonic frequency to circumvent broad-spectral effects. We found one significant positive cluster (p < 0.031;Fig. S1), with a similar pattern of increase in power ~ISF, and analogous broadband effects at later time points.

Subsequently, the cluster was qualitatively separated into a somatosensory and a frontal cluster. We found one significant cluster in the frontal region (p = 0.003,Fig. 2D), and three significant clusters in the somatosensory region (p < 0.040,Fig. 2C). The time–frequency profile of the clusters in both regions matched closely; but in the frontal cluster, early effects at higher frequencies were not significant.

**Fig. 3.**
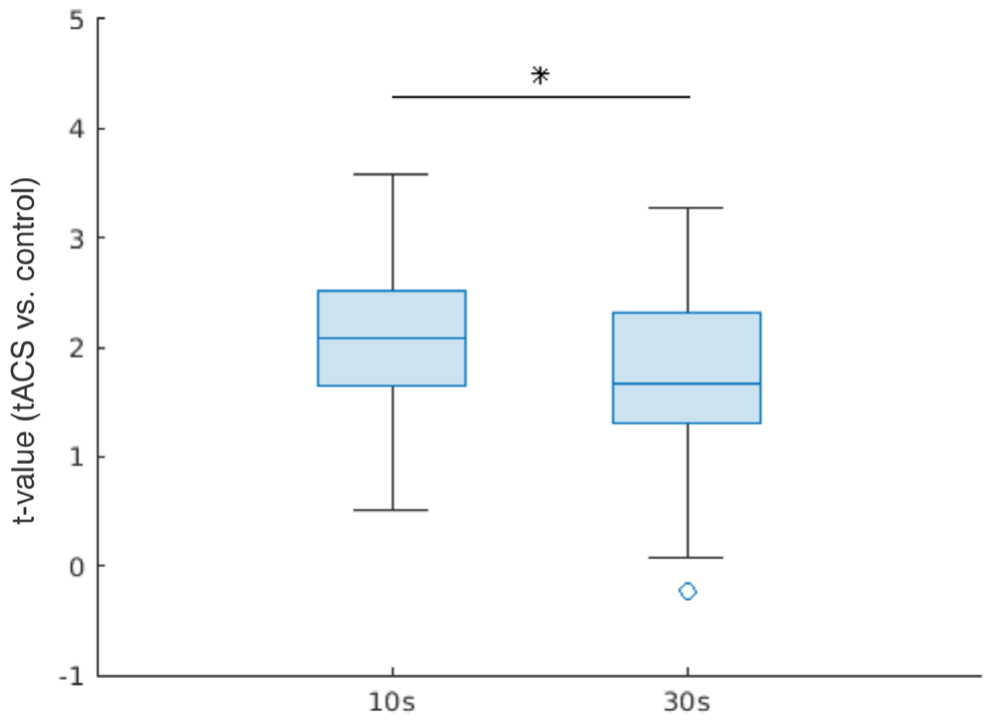
Modulation of power in 10s & 30s blocks. Boxplot shows t-values signifying the difference in Δpower between tACS and control conditions for the respective stimulation blocks. t-values were averaged within the significant cluster shown inFigure 2A. *p < 0.05.

To investigate the effect of train duration on power, we compared Δpower at ISF between 10 s and 30 s blocks (Fig. S2shows Δpower of individual participants). We found that the 10 s block produced greater increases in power than the 30 s block (p = 0.020;Fig. 3).

### 3.3 Binning analysis of stimulation trains

**Fig. 4.**
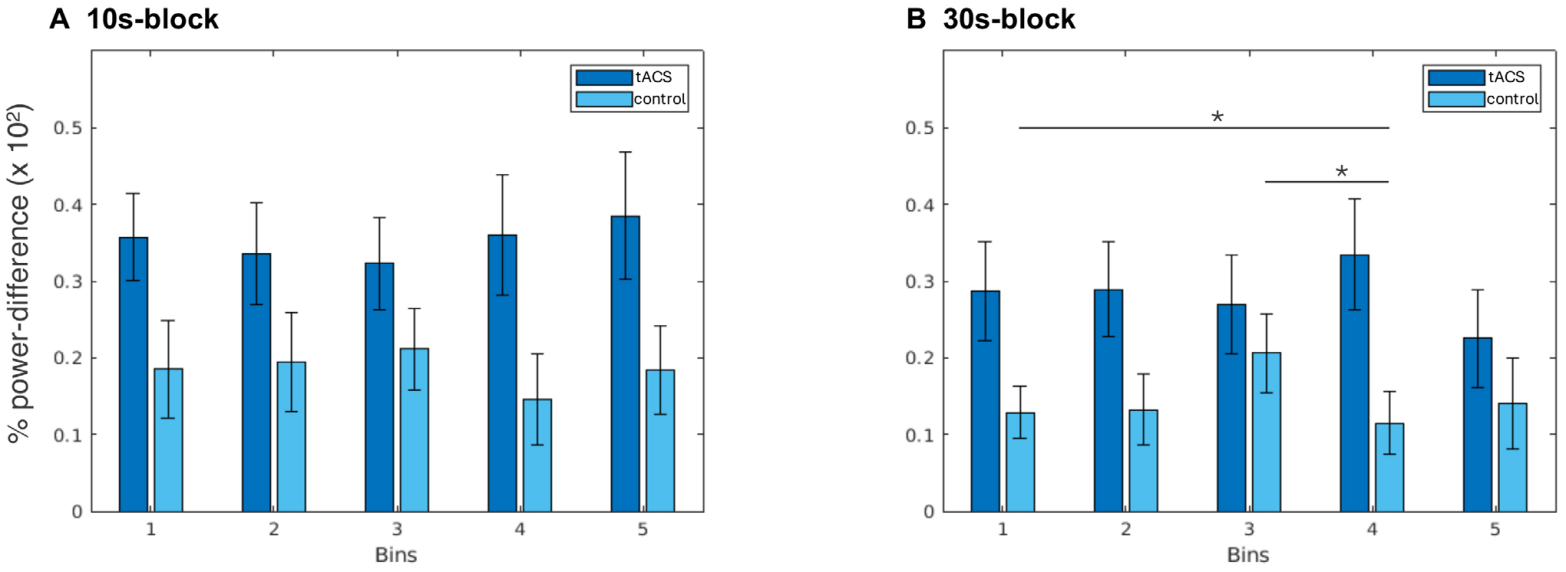
Modulation of power through the duration of the experiment. (A) Bars represent the percentage of power-difference at ISF (relative to baseline) for the 10 s block. Dark blue bars indicate power-difference in tACS, whereas light blue bars indicate power-difference in control. Error bars indicate standard deviation. Bin number increases with time on task. (B) Same as (A), but for the 30 s block. *p < 0.05.

In the 10 s block, there was no main effect of “Bin” in tACS (Χ^2^= 7.50, p = 0.112) or control conditions (Χ^2^= 7.93, p = 0.094;Fig. 4A). Similarly, in the 30 s block, there was no main effect of “Bin” in the tACS condition (Χ^2^= 6.83, p = 0.145), but a significant main effect of “Bin” (Χ^2^= 15.00, p = 0.005;Fig. 4B) in the control condition. A pairwise*post hoc*Conover test with Bonferroni correction showed a significant difference between the first and fourth bins (p = 0.016) and the third and fourth bins (p = 0.036).

### 3.4 Exploratory analysis of rhythmic power modulations

**Fig. 5.**
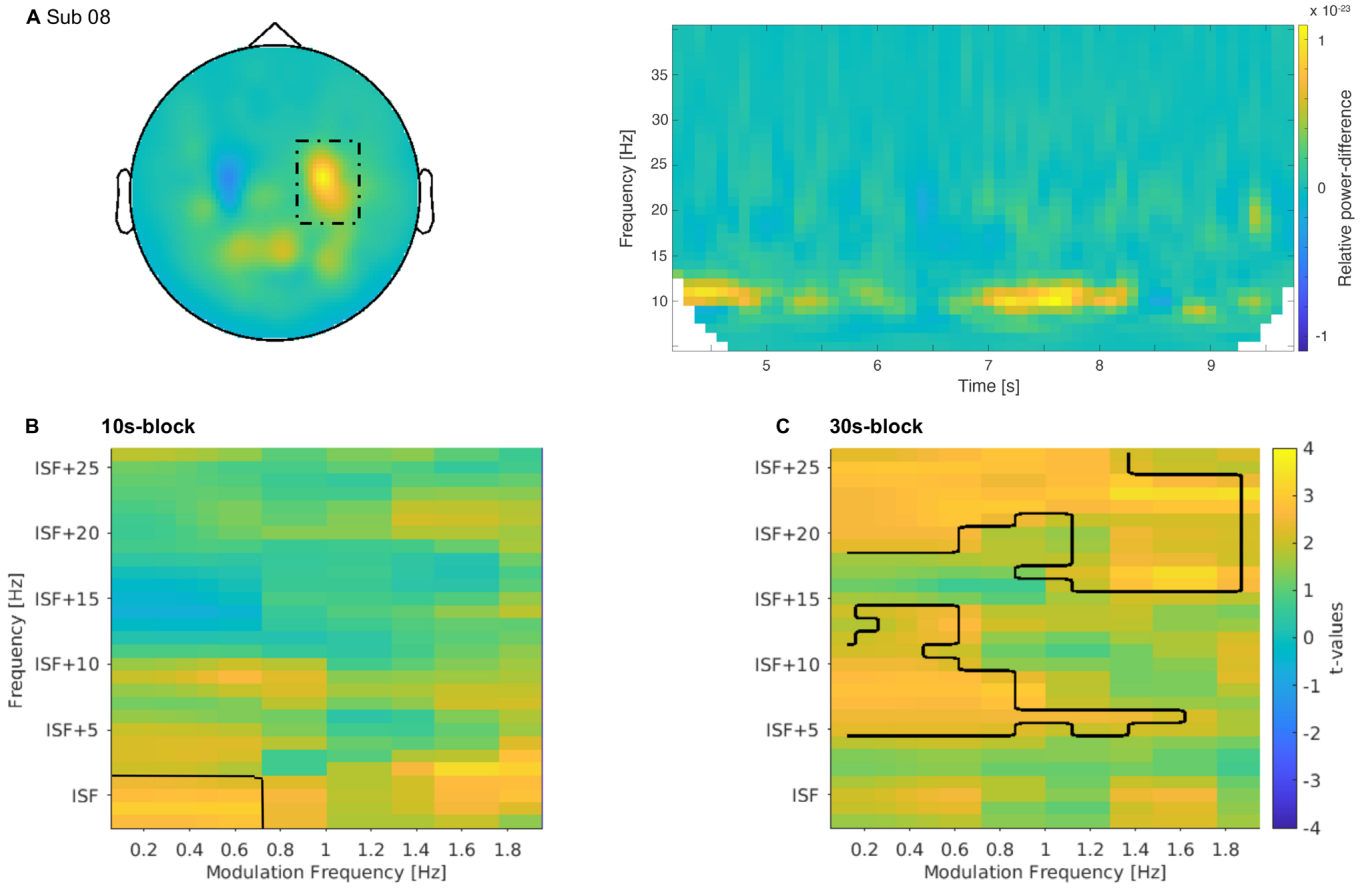
Exploratory analysis: Rhythmic fluctuation of power. (A) Exemplary demonstration of slow fluctuation of power in a single participant. The left panel shows sensor-level topography of power change (relative to previous trial) at ISF±1. Dotted black outline indicates the somatosensory region of stimulation. The right panel shows TFR of power averaged over channels within the dotted outline. (B) TFR of t-values on source level. t-values signify the difference in modulatory power between tACS and control conditions for the 10 s block. X-axis represents the modulatory frequencies. Y-axis represents the canonical frequencies and ranges from “ISF-2” to “ISF+26” Hz. We found one significant positive cluster (outlined in black). (C) Same as in (B), but for the 30 s block. We found two significant clusters (outlined in black). TFRs (in B & C) were averaged across channels in the significant cluster (inFig. 2A) for the respective stimulation blocks.

We observed systematic fluctuations of alpha power with time in several participants (Fig. 5A); hence, we statistically evaluated whether the magnitude and/or regularity of these fluctuations is modulated by tACS. We found one significant positive cluster in the 10 s block (p = 0.031,Fig. 5B), which spanned from 0.1 to 0.75 Hz for the modulation frequency and between ‘“ISF±2” Hz for the power. For the 30 s block, we found two significant clusters (p = 0.006 and p = 0.011,Fig. 5C). One cluster ranged from 0.1 to 1.75 Hz for the modulation frequency and from “ISF+16” to “ISF+26” Hz for the power. The other cluster ranged from 0.1 to 1.5 Hz for the modulation frequency and from ISF+5” to “ISF+14” Hz for the power. In general, the modulatory power appears to be enhanced with tACS compared with the control condition.

## 4 Discussion

The heterogeneous effects of tACS are an ongoing challenge in the field of non-invasive brain stimulation. Our study was motivated by the lack of uniformity in stimulation parameters, which may in turn explain the heterogeneity in reported effects. To study the influence of stimulation duration, we intermittently applied tACS for short periods of 10 s and 30 s during simultaneous MEG. Our results demonstrate that short-period tACS enhanced alpha power at individual stimulation frequency (ISF) in the somatosensory and frontal regions. Blocking transcutaneous stimulation via a topical anaesthetic did not hinder the modulation of power. Notably, short stimulation trains (10 s) produced a greater increase in alpha power than long stimulation trains (30 s).

Studies using short trains (8 s) of tACS to the parieto-occipital region have reported an increase in power (Stecher et al., 2021;Vossen et al., 2015). Whereas, the only study to administer intermittent tACS (6 s trains) to the somatosensory cortex did not find any changes in alpha power (Sliva et al., 2018). On the contrary,Gundlach et al. (2017)showed a decrease in alpha amplitude following 5 min of tACS to the somatosensory cortex. Our results stand in contrast to both accounts, demonstrating the ability of tACS to enhance somatosensory alpha power. Discrepancies between our results and the others’ may stem from variations in stimulation site and electrode placement, as the referenced studies used different setups: attaching electrodes to left S1 or bilaterally above left and right S1.

The power enhancement observed in our study spanned bilaterally over the somatosensory cortex and, surprisingly, it extended up to the frontal cortex. Similarly,Kasten et al. (2019)applied tACS over the parieto-occipital cortex and reported a widespread increase in alpha power across parieto-occipital, temporal, and frontal regions. Most studies only assess data from a selected region of interest; thus, little is known about dispersion of effects. New evidence suggests that tACS improves posterior-to-frontal alpha connectivity, implying that the widespread power increase might be due to altered network dynamics (Ahn et al., 2019;Clancy et al., 2022;Fuscà et al., 2018). Adding further credibility to this interpretation, we did not observe a congruent dispersion of electric fields in the current simulations. In general, our finding provides new insights into the spatial extent of tACS-induced after effects.

Further, we found that the power increase induced by tACS extended beyond the alpha-band to the beta-band. This finding diverges from studies showing power modulation specifically at ISF (Helfrich, Schneider, et al., 2014;Zaehle et al., 2010). However, entrainment is a non-linear process and is not restricted to the frequency of stimulation. The frequency and intensity at which an entrainment is successful are characterised by a triangular region of synchronisation, known as the “Arnold Tongue” (Pikovsky et al., 2002). For example, a neural oscillator with a peak in the beta frequency (~20 Hz) can be entrained by a 10 Hz rhythm (1:2 Arnold Tongue) and*vice versa*. Also, an increase in beta power can be explained by cross-frequency phase synchronisation. A similar 1:2 relationship for 10 Hz alpha and 20 Hz beta has been demonstrated extensively (Carlqvist et al., 2005;Nikulin & Brismar, 2006;Palva et al., 2005;Palva & Palva, 2018).

Power enhancement in beta frequencies persisted even after correcting individuals’ TFRs by aligning ISF with its first harmonic frequency. This shows that the effect was not simply a result of spectral smearing. However, the power increase observed at higher frequencies might be driven by non-sinusoidal features of the somatosensory alpha rhythm (Cole & Voytek, 2017;Donoghue et al., 2022). We found power increase for harmonic frequencies in the somatosensory region, but less so in the frontal region. Stronger alpha band power and greater deviation from sinusoidality coincide with stronger harmonics in the beta band (Donoghue et al., 2022;Schaworonkow, 2023). This might suggest that there is greater contamination of genuine beta-band activity in the somatosensory region due to its arc-shaped waveform. However, the concomitant changes in beta power and higher frequencies must be interpreted with caution, as the impact of alpha-tACS on other frequency bands and waveforms is grossly understudied.

Notably, we found 10 s trains showed a larger increase of alpha power than 30 s trains. Research employing transcranial direct current stimulation (tDCS) has revealed similar non-linear effects (Ho et al., 2016;Mosayebi Samani et al., 2019). For example, 13 min of tDCS increased cortical excitability, whereas applying it for 26 min attenuated excitability (Monte-Silva et al., 2013). Likewise, lower current intensities induced larger increases in alpha power than higher current intensities (4–6 mA) (De Koninck et al., 2021). Longer stimulation times and higher current intensities might be counteracted by metaplasticity, which prevents over-excitation and over-inhibition of neural populations (Abraham, 2008). Thus, further research is required to understand the dose-dependent effects of tACS, especially in the somatosensory cortex.

Additionally, we split post-stimulation trials into equal-sized time bins to evaluate power modulation as a function of the number of tACS trains administered. We did not find statistically significant power-difference across all bins for both 10 s and 30 s tACS blocks. This finding reiterates that short trains of stimulation produce concurrent and consistent changes in alpha power. We found some variability in power modulation in the 30 s control block, which may reflect a natural variation in alpha power due to increased mental fatigue (Boksem et al., 2005;Oken et al., 2006).

Additional exploratory analyses showed slow fluctuations in alpha power (~0.4 Hz), which are enhanced with tACS compared with control. It appears that tACS either facilitates rhythmicity in power (i.e., improves the occurrence of burst-like activity), or merely enhances the amplitude of the oscillation relative to control. In either case, we would observe a facilitation of modulatory power at the frequency of stimulation. While our analysis is not sufficient to delineate the nature of the observed changes, it presents an exciting opportunity for further study. The slow fluctuations of power might be driven by bodily rhythms (e.g., heartbeat and respiration) (Kluger et al., 2021;Yuan et al., 2013). However, this interpretation warrants caution, as we did not measure ECG or respiration.

ISF (estimated at the beginning of each session) and alpha peak frequency (during the experiment) did not match for two-thirds of our participants (Kasten et al., 2019;Stecher & Herrmann, 2018). Our target was somatosensory alpha oscillations, but since we estimated ISF from sensor-level data, it is possible that our estimates were contaminated by the dominant occipital alpha rhythm (Schaworonkow & Nikulin, 2022). Additionally, peak frequency has been shown to shift over time, further complicating its estimation (Benwell et al., 2019;Haegens et al., 2014). Eight participants had a matching peak frequency in the 10 s block, whereas 10 participants had a matching peak frequency in the 30 s block. Therefore, the larger effect on power seen in the 10 s block is not attributable to disproportionate shifts in peak frequencies between the two stimulation blocks. Another limitation is loss of the first ~4 s post-stimulation due to artefacts from the stimulation device. As entrainment echoes vanish a few milliseconds after stimulation offset, we could not assess the evidence for or against the entrainment hypothesis. Therefore, the role of plasticity and entrainment in producing the observed effects remains unclear. Future studies should consider employing the remote input available in DC-Stimulator PLUS (NeuroConn, Germany) to control the stimulation signal on a trial-by-trial basis.

In this study, we reiterate the potential of intermittent short-period tACS to induce oscillatory power changes. Importantly, we established that 10 s of tACS is sufficient for our intended use in a prospective study, and that longer stimulation periods are not a prerequisite for modulating power. Taken together, our findings strongly advocate the application of tACS to further our understanding of brain and behaviour.

## Supplementary Material

Supplementary Material

## Data Availability

The raw MEG data and scripts are available upon request.
