# Supplementary material for: Modulating somatosensory alpha oscillations using short-period transcranial alternating current stimulation
Source: Imaging Neurosci (Camb). 2025 Apr 7;3:imag_a_00531. doi: 10.1162/imag_a_00531 (PMC12319803; doi:10.1162/imag_a_00531)
Supplement: Supplementary Material [file imag_a_00531-supp.pdf]

Supplementary Material

Table S1: Individual Stimulation Frequency (ISF) and Alpha Peak Frequencies

Subject	*ISF (Hz)*	*10s-tACS (Hz)*	*30s-tACS (Hz)*	*10s-control (Hz)*	*30s-control (Hz)*
1	8	9/10	9	11	10
2	10	10	10	10	9
3	12	8/10	9	11	9
4	9	9	9	9	9
5	14	10	8	11	9
6	9	10	9	10	10
7	10	13	10	12	8
8	10	10	10	9	8
9	9	9/10	10	11	10
10	7	9	9	9	9
11	11	12	11	11	11
12	13	8	12	9	13
13	10	11	12	12	11
14	11	11	8/11	9	12
15	11	12	not available	12	12
16	8	9	9	8	10
17	9	10	10	9	10
18	12	12	12	12	12
19	10	9	9	10	8
20	10	11	11	11	11
21	10	10/11	10	9	10
22	10	9	9	9	10
23	9	10	9	10	8
24	11	11	11	11	11

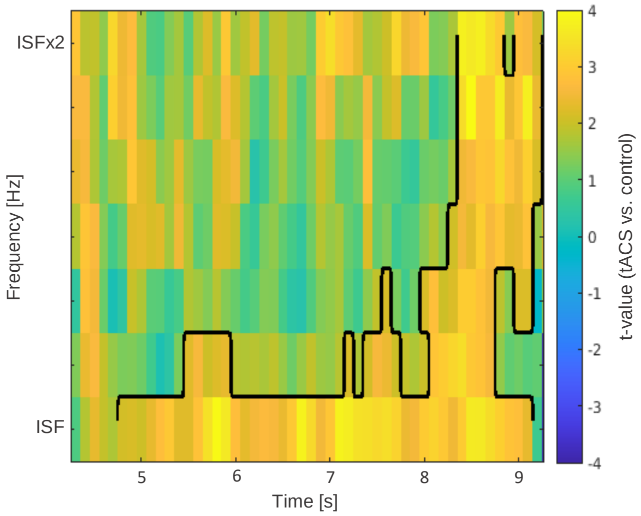

Figure S1: Control Analysis

On uniformly shifting the TFR to align ISFs to 10 Hz, the harmonic frequencies are misaligned. To circumvent broadband-spectral increase in power and thereby delineate harmonic effects, we recomputed the analysis in Fig. 2B. In the control analysis, we restricted the frequency range between ISF and its first harmonic frequency. Hence, for an ISF of 10 Hz, the frequencies ranged from 10 to 20 Hz, and for an ISF of 7 Hz, from 7 to 14 Hz. Five intermediate frequencies were selected between ISF and the first harmonic of ISF. Y-axis ranges from 'ISF' to 'ISFx2' Hz (first harmonic frequency). t-values were averaged over channels in the significant cluster shown in Fig 2A. We found one significant positive cluster (outlined in black).

A 10s-block

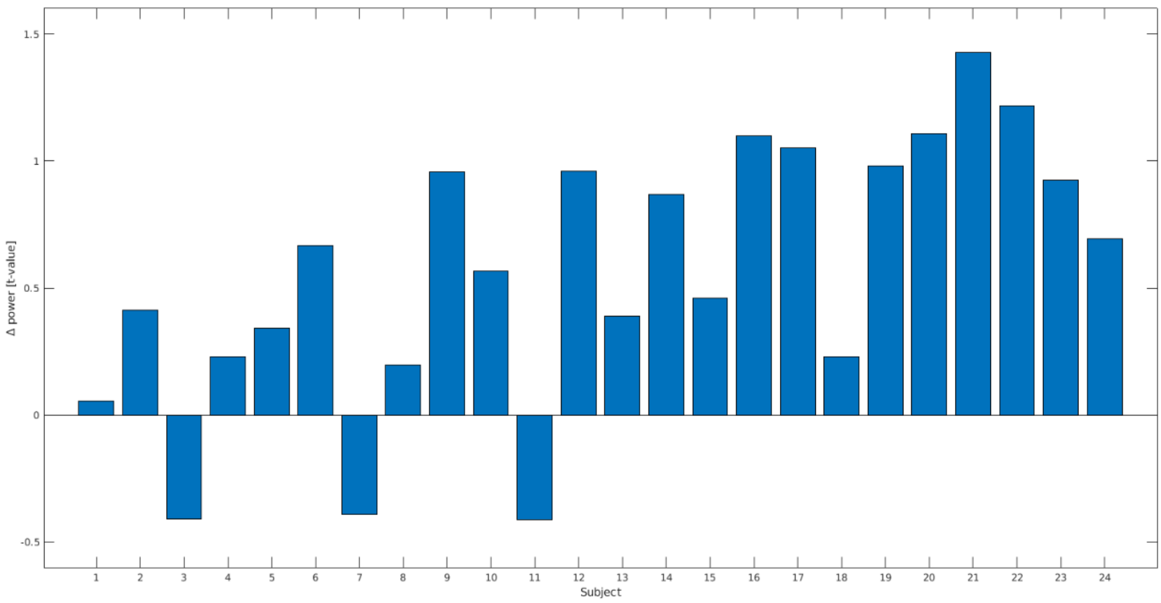

B 30s-block

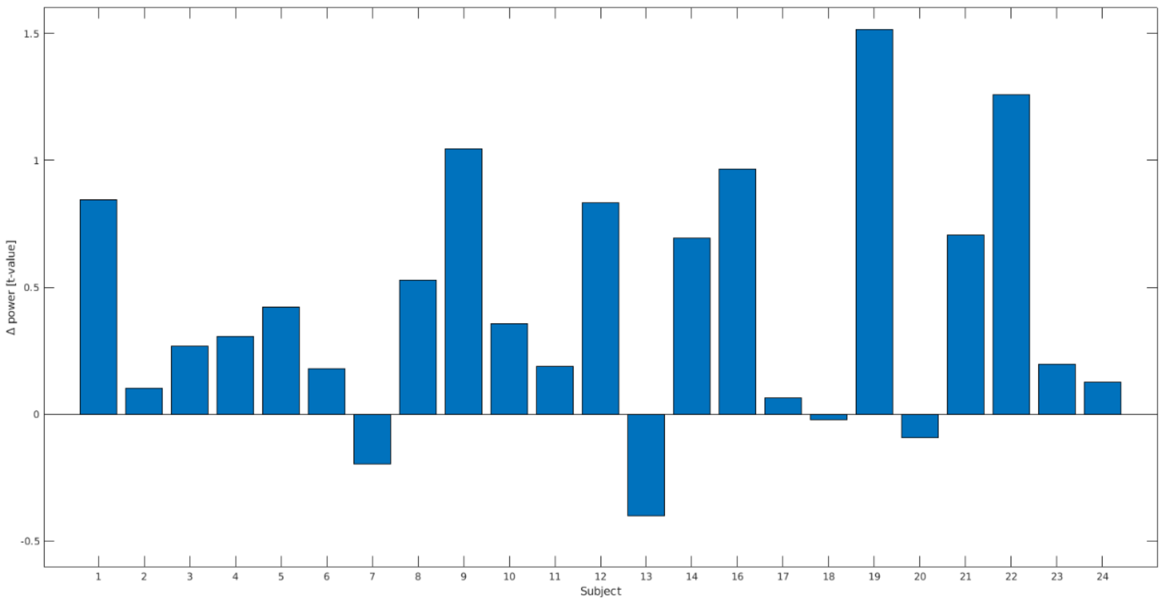

Figure S2: Inter-individual variation in response to tACS
A) Bar graph illustrates Δpower for the 10 s-tACS block. Δpower (at ISF) was averaged over channels in the significant cluster shown in Fig. 2A, and over time. Each bar represents a single subject. **B)** Same as in A), but for 30 s-tACS block.
